# Apicobasal transferrin receptor localization and trafficking in brain capillary endothelial cells

**DOI:** 10.1186/s12987-022-00404-1

**Published:** 2023-01-09

**Authors:** Simone S. E. Nielsen, Mikkel R. Holst, Kristine Langthaler, Sarah Christine Christensen, Elisabeth Helena Bruun, Birger Brodin, Morten S. Nielsen

**Affiliations:** 1 Department of Biomedicine, Faculty of Health, Aarhus University, 8000 Aarhus C, Denmark; 2 CNS Drug Delivery and Barrier Modelling, University of Copenhagen, Copenhagen, Denmark; 3 Translational DMPK, H. Lundbeck A/S, Copenhagen, Denmark; 4 Pathology & Imaging, Novo Nordisk, Måløv, Denmark; 5 Department of Pharmacy, Faculty of Health and Medical Sciences, University of Copenhagen, 2100 Copenhagen, Denmark

**Keywords:** Transferrin receptor (TfR), Intracellular trafficking, Apicobasal polarity, Brain drug delivery, Blood–brain barrier, Brain endothelial cells, Expansion microscopy

## Abstract

The detailed mechanisms by which the transferrin receptor (TfR) and associated ligands traffic across brain capillary endothelial cells (BECs) of the CNS-protective blood–brain barrier constitute an important knowledge gap within maintenance and regulation of brain iron homeostasis. This knowledge gap also presents a major obstacle in research aiming to develop strategies for efficient receptor-mediated drug delivery to the brain. While TfR-mediated trafficking from blood to brain have been widely studied, investigation of TfR-mediated trafficking from brain to blood has been limited. In this study we investigated TfR distribution on the apical and basal plasma membranes of BECs using expansion microscopy, enabling sufficient resolution to separate the cellular plasma membranes of these morphological flat cells, and verifying both apical and basal TfR membrane domain localization. Using immunofluorescence-based transcellular transport studies, we delineated endosomal sorting of TfR endocytosed from the apical and basal membrane, respectively, as well as bi-directional TfR transcellular transport capability. The findings indicate different intracellular sorting mechanisms of TfR, depending on the apicobasal trafficking direction across the BBB, with the highest transcytosis capacity in the brain-to-blood direction. These results are of high importance for the current understanding of brain iron homeostasis. Also, the high level of TfR trafficking from the basal to apical membrane of BECs potentially explains the low transcytosis which are observed for the TfR-targeted therapeutics to the brain parenchyma.

**Supplementary Information:**

The online version contains supplementary material available at 10.1186/s12987-022-00404-1.

## Background

While the mechanisms of iron transport across the physiological barriers at the gut and placenta have been intensively studied for decades, the detailed mechanisms of brain iron transport and regulation are still not fully elucidated [1]. These mechanisms involve trafficking of the Transferrin receptor (TfR) and its natural ligand, iron-binding Transferrin (holo-Tf), with still no unified understanding of the intracellular trafficking pathway [2]. Interestingly, TfR-targeted brain drug delivery using the “trojan-horse” like strategy of receptor-mediated transcytosis (RMT) for specific delivery across blood–brain barrier (BBB) is considered one of the most promising approaches for drug delivery of biotherapeutics to the brain [2]. Massive research effort within academia and biotech aims to contribute with such valuable treatment methods for the continuously growing number of patients with disorders of the CNS [3, 4]. Although it may be possible to penetrate the BBB using the TfR-mediated trafficking [5], still no effective transport strategy which can be used for a large spectrum of biopharmaceutical drugs across the BBB has been reported [6, 7]. One limitation is a major knowledge gap concerning the physiology of brain iron homeostasis, which hampers the design of an optimal therapeutic TfR targeting construct for RMT across the BBB. Of high importance, the BECs harbor undisclosed regulatory trafficking mechanisms, including the endosomal system [8], and TfR sorting [2, 9], though difficult to study due to the flat morphological nature, abrogating conventional trafficking studies.

BECs differ from the peripheral vascular endothelial cells in both morphology, structure, and functional characteristics [10], and accordingly may not mirror the mechanisms reported for other endo- or epithelia [8, 11]. The developmental specialization enables a highly selective transport, maintained in a close interplay with cells of the neurovascular unit, including pericytes, myocytes, astrocytes, and neurons [12–15]. Maintenance of CNS microenvironmental homeostasis depends on the apicobasal polarity developed in BECs, which sets the stage for receptor localization on either the blood (apical) or brain (basal) localized membranes [9]. TfR localization has been reported on the apical surface of BECs back in 1984 [16], initiating the hypothesis of TfR being a part of a mechanism for iron transport to brain tissues [16], with the iron-binding complexes hypothesized to undergo transcytosis from the apical to basal membrane [17–19]. Nevertheless, a simpler trafficking circuit seems to have several flaws [2, 20]. These include; the need of a mechanism to provide BECs with iron for their own needs; the need of a mechanism inducing dissociation of the TfR-holo-Tf complex at the basal plasma membrane, and importantly; a mechanism for regulating iron uptake and maintenance of brain iron homeostasis [20]. Furthermore, a simple transcytosis trafficking circuit of the TfR-holo-Tf complex is contradictory to subsequent findings of higher import rates of iron than Tf to the brain [21–23], engendering the theory of TfR recycling. This recycling theory encompasses TfR-ligand dissociation due to endocytic acidification, iron release from endosomal vesicles through divalent metal transporter 1 (DMT1) [1, 24] and/or incorporation in the storage protein ferritin [25]. Following iron release through DMT1, iron is described to be released to the extracellular environment through the iron exporter ferroportin, requiring ferroxidase activity [26], while TfR is believed to recycle to the plasma membrane [2]. This intracellular trafficking mechanism of TfR has been debated for decades [1, 2, 24, 27–29], but based on the current research within TfR trafficking and brain drug delivery, it cannot be ruled out whether both transcytosis and/or re-cycling mechanisms are involved [2].

Noteworthy for the plethora of studies within TfR and the BBB, the majority have considered only apical TfR localization in order to accept the system as accessible for brain drug delivery, and convincingly reported TfR expression in BECs across numerous species and models [2, 4, 30, 31]. Investigations of basal TfR expression, however, remain limited [32], despite previous studies suggesting TfR to be altered apically, intracellularly, and basally in BECs, dependent on the brain iron status [20]. Investigation of such basal TfR localization and potential brain-to-blood trafficking may add valuable knowledge to our understanding of brain iron homeostasis as well as future approaches of designing TfR-targeted therapeutics not susceptible for reuptake at the basal membrane.

In this study, we aimed to distinguish between neosynthesized TfR and endocytosed TfR directed towards endosomal sorting, which we hypothesized to traffic differently. We therefore performed the investigations under normal- and brefeldin A (BFA) conditions, the later inhibiting protein transport between endoplasmic reticulum (ER) and Golgi, retaining neosynthesized TfR. To address the technical challenge and demand of improved methods to distinguish the close proximity of the apical and basal membranes of BECs [9], we used expansion microscopy (ExM) [33]. This technique enabled the use of conventional high resolution confocal microscopy for imaging primary porcine BECs (pBECs) in a non-contact co-culture (NCC) in vitro BBB model [34]. We designed ExM with a six-time increase in imaging resolution allowing differentiation of the apical and basal membranes. Overall, the findings confirm basal TfR and bi-directional apicobasal trafficking in BECs, providing a new understanding of TfR trafficking, relevant for brain iron homeostasis and future RMT brain drug delivery approaches.

## Materials and methods

### Reagents

All reagents and chemicals were purchased from Sigma-Aldrich (Rødovre, Denmark), unless otherwise stated.

### Cell cultures and in vitro BBB model establishment

Astrocytes were purified from 1–2 days old Sprague–Dawley rats, and brain microcapillaries were purified from 5–6 months old pigs. Selective cultures of pBECs were established in a non-contact co-culture (NCC) in vitro BBB model as previously described in detail [34]. Following primary cell purifications, astrocytes were cultured in poly-L-Lysine pre-coated 12-well plates in DMEM low glucose supplemented with 10% fetal bovine serum (FBS), penicillin (100 U/mL), and streptomycin (100 µg/ml) three weeks prior to NCC establishment. Porcine brain microcapillaries were seeded on type IV collagen- (150 μg/mL) and fibronectin (50 μg/mL) coated T75 flasks using DMEM-F12 supplemented with 10% plasma-derived serum (PDS) (First Link, Wolverhampton, United Kingdom, UK), penicillin (100 U/mL), streptomycin (100 µg/ml), and heparin (15 U/mL) as growth media. For the first four days in culture, pBEC were selected using puromycin (4 μg/mL). At 70% confluency, cells were passaged with Trypsin/EDTA (2.5% trypsin, 0.1 nM EDTA in PBS) and seeded on type IV collagen (500 μg/mL) and fibronectin (100 μg/mL) coated Transwell inserts (12 mm, 0.4 μm pore polycarbonate membrane, cat. no: 3401, Corning, Kennebunk ME 04043, USA) at a density of 1.1 10^5^ cells/insert. pBECs were co-cultured with astrocytes in the basal chamber in culturing media without serum. To further induce the barrier development, both chambers were supplemented with the differentiation factors hydrocortisone (550 nM), 8-(4-chlorophenylthio)-adenosine-3ʹ,5′-cyclic monophosphate (250 μM), and RO-201724 (17.5 μM) 1–2 days prior to experiments. The integrity of the model was validated by measurements of transendothelial electrical resistance (TEER) using an EndOhm-12 measurement device (World Precision Instruments), with values > 1000 Ω cm^2^ accepted for experiments. Furthermore, the expression of tight and adherens junctional proteins were validated from immunocytochemistry according to the procedures described in the ‘*Immunofluorescence staining*’ section. Prior to all experiments, Transwell inserts with cultured pBECs were transferred to a new 12-well plate without astrocytes, washed two times with PBS, incubated with experimental buffer and allowed to rest at 37 °C, 5% CO_2_ before further treatment.

### Quantitative PCR

Purification of mRNA was performed by scraping NCC established pBECs off the Transwell filter inserts using a 200 μl pipette tip and 300 μl lysis buffer using an RNA purification kit according to the protocol prescription (740955.10, Macherey–Nagel). Purified mRNA was transcribed into cDNA by reverse transcription polymerase chain reaction (RT-PCR) and the TaqMan Reverse Transcription Method (N8080234, Invitrogen), followed by quantitative PCR (qPCR) analysis and application of a dye-based detection technique. The relative gene expression level was calculated by the 2^−ΔΔCt^ method (Livak & Schmittgen, 2001) and normalized to the geometrical mean of the housekeeping genes β2M, Ribosomal Protein L4, TATA-Box Binding Protein, and Hypoxanthine Phosphoribosyltransferase 1.

### Western blotting

Samples were mixed from protein sample with ExB lysis buffer (1% Triton X-100, 150 mM NaCl, 2 mM MgCl_2_, 2 mM CaCl_2_, complete mini protease inhibitor cocktail (Roche)) (65%), 0.2 M DTT (10%), and NuPAGE LDS sample buffer (25%) (Invitrogen, NP0007), heated to 95 °C for 5 min. Samples were loaded to 4–12% bis–tris gels next to SeeBlue™ Plus2 pre-stained protein standard as protein marker in a running buffer prepared from 20 × NuPAGE MES buffer, ddH2O, and NuPAGE antioxidant. Samples were run for 45 min at a constant of 140 V. Gels were afterwards transferred for dry-blotting using Novex iBlot dry blotting system (Invitrogen, cat#IB24001), and blocked with Tris-buffered Saline (TBS), 0.1% tween-20 and 5% milk for 1–2 h at RT. The blots were incubated ON with primary antibodies at 4 °C on a rolling table, and on the following day washed in 0.1% Tween-20 in PBS, followed by incubation with secondary antibodies for 1 h at RT. After washing in 0.1% Tween-20 in PBS, the blots were developed using ECL development reagent, and detected using an iBright 1500 (Invitrogen) chemiluminescence imager. Specifications of the applied antibodies are found in Additional file 1: Table S1.

### Endothelial polarization by bi-directional transport experiments of Rhodamine 123

The experimental setup, including solubility, concentration considerations and the standard curve interval were determined based on previous thermodynamic solubility data [35, 36]. BECs were equilibrated with transport buffer of DMEM-F12, no phenol red (Gibco, Thermo Fischer Scientific), penicillin (100 U/mL), streptomycin (100 µg/ml), and heparin (15 U/mL) for 2 h at 37 °C, 5% CO_2_ before further treatment. Applied volumes were 400 μl in the apical chamber and 800 μl in the basal chamber. To investigate the apical-to-basal (A-B) and basal-to-apical (B-A) transport of the fluorescent tracer dye Rhodamine 123 (R123) with specificity for the ABC efflux transporter p-glycoprotein (P-gp, ABCB1), the compound was added either the apical or basal compartments in amounts of 100 or 200 UL, respectively, with resulting final concentrations of 3 μM or 30 μM. Additionally, DMSO was spiked in the opposite chamber to achieve final DMSO concentration of 0.25% in both chambers. R123 was allowed to incubate at 37 °C, 5% CO_2_ up to 120 min with a circular rotation of 100 rpm and an orbit of 3 mm. Receptor aliquots were collected from the apical and basal chambers (50 and 100 μl) at 0, 15, 30, 60, 90, and 120 min, and the volumes replaced with equivalent volumes of buffer. Donor samples were collected at zero and 120 min to guarantee steady state conditions were maintained throughout the experiment. The zero minutes donor samples were prepared from separate chambers mimicking the donor concentrations, avoiding elimination of material from measured samples. For measurement of material within cells and filters, these were washed 3 times with ice cold buffer, removed to Eppendorf tubes and added 0.1% Triton X-100 in MilliQ H_2_O to lyse cells and allow material to be analyzed. Samples with R123, controls and standard curve of 9 standards with increasing concentration of R123 (0.01 to 1 μM) were pipetted onto a 96-well fluorescence plate and analyzed using a microplate reader and excitation and emission wavelengths of 485 and 520 nm, respectively. All samples were diluted with buffer to fit the range of the standard curve.

### Data treatment

The apparent permeability coefficients (P_app_) for R123 were calculated from the fluorescent readout using the following equation:

1 $${P}_{app }= \frac{{J}_{ss}}{{C}_{0}}= \frac{{Q}_{t}}{{C}_{0}*A}$$

J_ss_ represents the steady state flux (mol cm^− 2^ s^− 1^), with the fluxes of R123 calculated as the slope of the linear part of the curve from plots of Q_t_, the accumulated receiver amount transported at the given time t (min), against time t (min). C_0_ represents the start donor concentration of R123, and A equals the area of the filter membrane of the Transwell insert (1.12 cm^2^). Efflux ratios were established from the ratio between P_app_ in the two directions, A-B and B-A, as presented in following equation:

2 $$Efflux ratio= \frac{{P}_{app B-A} }{{P}_{app A-B} }$$

The efflux ratios were used to determine transporter involvement of R123 across the pBEC monolayer. An efflux ratio > 2.5 was set as the threshold to indicate active efflux of R123.

### Immunofluorescence staining (IF)

pBECs were permeabilized using 0.1% Triton-X in PBS for 10 min, RT. Afterwards, the filters were washed in PBS and blocked with 2% BSA in PBS for 30 min at RT. Primary and secondary antibodies were applied according to the manufacturer’s descriptions, with the specific descriptions noted in Additional file 1: Table S1, and the membranes in between and after incubations washed in PBS. For nuclei staining, cells were washed twice in ddH_2_O, RT, and incubated with 0.125 μg/mL Hoechst stain solution (Sigma-Aldrich) in ddH_2_O for 10 min, RT. Finally, membranes were mounted on SuperFrost microscope glass slides (Hounisen Laboratorieudstyr) using fluorescent mounting medium (Dako) and 1.5 mm cover slips (Thermo Fischer Scientific). The slides were left over night at RT in darkness and sealed with nail polish before imaging. For fixation of microcapillaries, these were transferred to a petri dish with cold, sterile PBS and centrifugated for 1 min at 250 g. The supernatant was carefully discharged and cold 4% PFA in PBS was added to the capillaries for 12 min. The microcapillaries were again centrifuged for 1 min at 700 rpm, washed twice with cold PBS for 5 min and aliquoted depending on density. For immunostaining, the capillaries were handled in Eppendorf tubes, in which washing was performed by adding 100 mL of solution, incubating for 10 min, centrifugate for 1 min at 250 g and discharging of the supernatant.

### Brefeldin a treatment in cellular assays

Brefeldin A (BFA, Sigma cat. No.: B6542), inhibiting vesicular transport between ER and Golgi, was included as a pre-treatment in several experiments using 10 μg/mL in culturing media 20 min prior to the cellular assay.

### Expansion microscopy (ExM)

BECs were fixed for 20 min using 4% paraformaldehyde in cytoskeleton buffer at RT, and afterwards washed 3 times with RT PBS. Filter pieces were cut free of the Transwell inserts and carefully placed on pieces of parafilm, and handled in droplets of the experimental solutions. Initially, permeabilization in freshly made 0.1% Triton X-100 in PBS for 10 min. Subsequently, cells were blocked in freshly made 2% BSA in PBS for 30 min. For immunomarking, primary antibodies were applied in a 5 times higher concentration than for normal IF, diluted in 2% BSA in PBS, and incubated for 1 h at RT. Filters were washed 3 times in PBS at RT before 30 min incubation in dark with secondary antibodies and Hoechst for nuclei marking, also applied in a 5 times higher concentration, and subsequently washed several times with PBS at RT. See Additional file 1: Table S1 for detailed information of applied antibodies and concentrations. (See Additional file 2).

Briefly explained, the expansion procedure consists of an anchoring step with a cross-linker, a gelation step to embed proteins in a swell-able acrylamide gel via the cross-linkers and an expansion step were water washing removes salt bridges between acrylamide polymers releasing and stretching the elastic acrylamide polymers [33]. Here expansion microscopy was performed as previously described [37, 38], using a modified gel recipe to achieve 6X expansion. The 6X expansion gel scale was found proportional fit for imaging using a spinning disc confocal Olympus imaging system with an UPlanSApo 60X, NA 1.20, water objective lens. This was based on the size of the 6 × expanded cell sample and the imaging field of view, which with the used imaging system enabled imaging of one entire cell, thus avoiding stitching of images. The cellular expansion procedure was initiated by cross-linking cells with 0.1 mg/mL Acryloyl-X in PBS for 30 min at RT, followed by several washes with PBS. The gelation procedure was performed by careful drying of filter pieces and placing them cell-side up on the parafilm piece, followed by adding ice cold droplets (40 μl) of gelation solution on top and finally placement of a #1.5 coverslip on top. The gelation solution was prepared from a monomeric 6X solution with final concentrations of 150 mM NaCl, 14% (w/w) acrylamide, 0.01% (w/w) N,N’-methylenebisacrylamide, and 14.3% (w/w) sodium acrylate in PBS, and concentrated stock solutions of ammonium persulfate (APS) and tetra-methyl-ethylene-diamide (TEMED), diluted in the monomeric solution to 0.2% (wt/wt). The gelation procedure was allowed for 30–60 min at RT. Carefully, the gel and coverglass were released from the parafilm underlayer, placed in digestion buffer with 0.5% Triton X-100, 0.8 M guanidine HCl in TAE buffer (40 nM Tris, 20 nM acetic acid, 1 mM EDTA) and Proteinase K (0.8 units/mL), and incubated for 45 min at 37 °C. Subsequently, specimens were placed in DI water for expansion ON, and on the following day water was exchanged every 30 min during 3–4 times. In the last water wash, Hoechst (1:20.000) was included for nuclei staining. The expanded specimens were cut to fit into a circular 35 mm Chamlide chamber (Live Cell Instruments, Cat. No.: CM-B-40) placed upon the 25 mm coverslip fitting the bottom of the magnetic holder. Excess water was removed, and the magnetic top was placed on top and preferentially immobilizing the gel, preventing the specimen from drifting during the microscopy session.

### TfR internalization and co-localization assay

Following establishment and validation of the BBB model, a pulse-chase study of receptor endocytosis was applied as previously described in detail [39]. Briefly, cells were cooled to 4 °C for 30 min in order to pause cellular activity, then primary anti-TfR antibody was applied to the apical or basal compartment, respectively, and the cells kept at 4 °C for further 90 min. Following cold incubation and surface receptor saturation with primary antibody, media was replaced with prewarmed antibody-free media, and cells incubated at 37 °C, 5% CO_2_ for 2, 10 or 20 min, respectively. Subsequent to the incubation times, filters were fixated and co-stained for intracellular markers using the described IF procedure.

### Immunocytochemistry transcellular trafficking assay

Primary anti-TfR antibody and secondary fluorescent antibody were spiked into the apical and basal compartment, respectively, and incubated at 37 °C, 5% CO_2_ for 45 min. Following antibody incubation, pBECs were fixated using 4% paraformaldehyde in PBS for 15 min, followed by 3 × 5 min washing in PBS. A semi-quantitative analysis was performed using spot detection in IMARIS software version 8.2 (Bitplane), with data presented as spots pr. cell and the apical and basal transcellular capability normalized between 0 and 100 for each experiment.

### Confocal microscopy and image processing

Confocal imaging was performed using an Olympus IX-83 fluorescent microscope with a confocal spinning disk unit (Yokogawa), Andor iXon Ultra 897 camera, and Olympus CellSens software. The specific objective lenses applied in the various experiments are denoted in the figure legends of the results, with all images captured as z-stacks with a step size of 0.31 μm. Pictures are presented as maximum-intensity z-stack projections unless other specific details noted in figure legends. Image processing and spot segmentation analysis was performed using IMARIS software version 8.2 (Bitplane).

### Statistics

All presented data are based on three independent experiments The statistical analyses and graphs were prepared using Prism (9.0) (GraphPad Software). The bar plots in graphs present mean values (±SEM), with Shapiro–Wilk test used for testing differences in standard deviations, and test of significant difference analyzed using t-test and 2way ANOVA with Sidak’s multiple comparison test and Tukey’s multiple comparison test, with *p < 0.05, **p < 0.01, ***p < 0.001 ****p < 0.0001.

## Results

### In vitro BBB model establishment and integrity

**Fig. 1:**
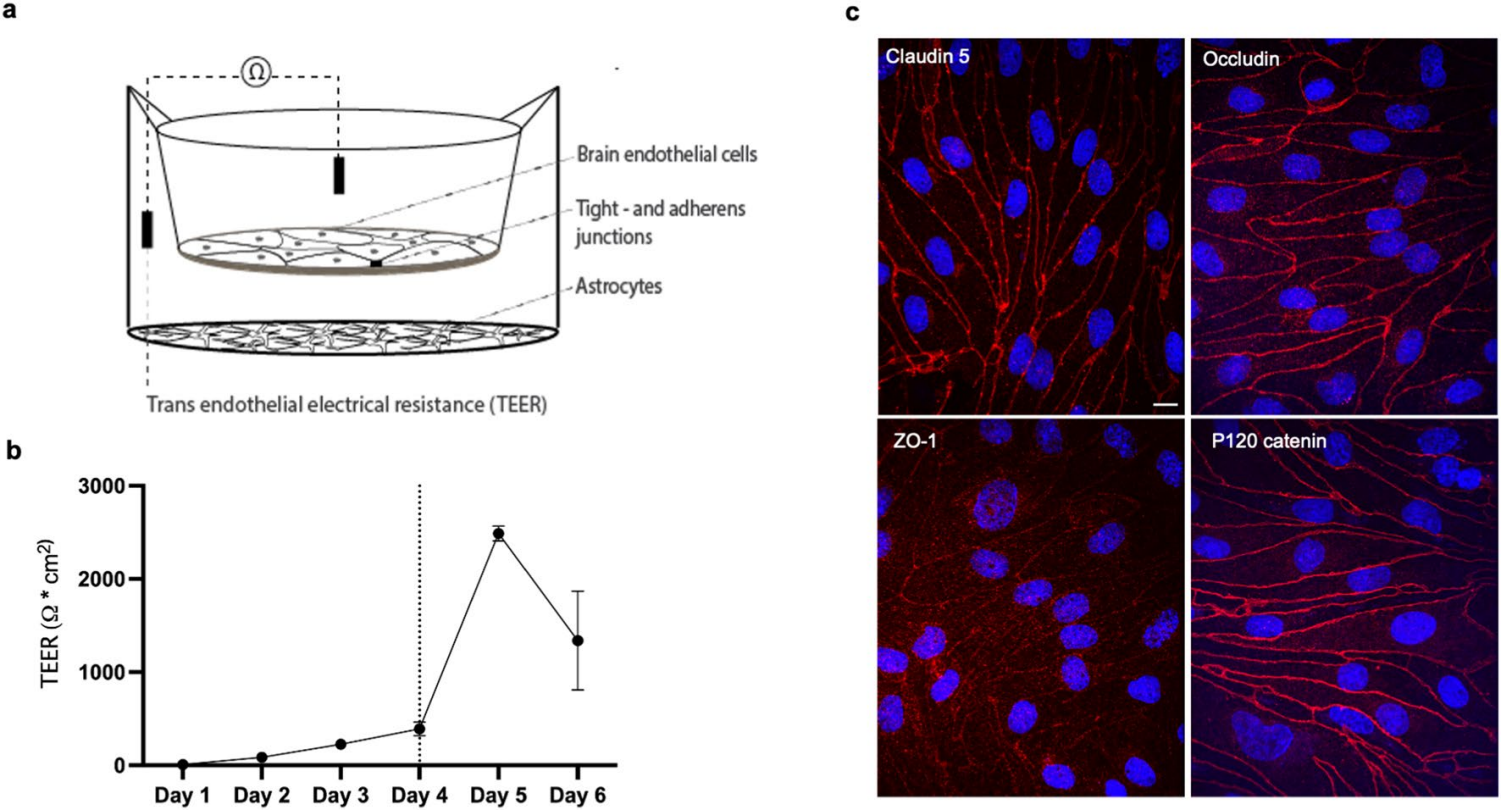
In vitro BBB model integrity. Schematic illustration of the applied non-contact co-culture (NCC) in vitro blood–brain barrier (BBB) model setup including primary porcine brain endothelial cells (pBECs) and astrocytes (**a**), with validation of the barrier integrity by transendothelial electrical resistance (TEER) (Ω cm^2^) presented as mean values (± SEM) and vertical line marking the day of inducement (**b**), and confocal microscopic imaging with 60 × magnification of immunofluorescent stainings (**c**) visualizing the tight- and adherens junctional (TJ and AJ) proteins claudin-5, ZO-1, occludin and p120 catenin (red), and Hoechst stain of nuclei (blue), with scale bar equal to 10 μm

For investigation of TfR membrane domain localization and intracellular trafficking, we established an in vitro co-culture model of the BBB as previously described in detail [34]. In this Transwell setup, pBECs were seeded on semipermeable membranes pre-coated with proteins of the basememnt membrane (fibronectin and collagen IV), with astroctes cultured in the bottoom compartment (Fig. 1a). Following barrier induction with in vivo differentiating factors, the barrier intigrity was validated from immunocytochemistry using confocal microscopy, with the endothelial barrier showing *in-vivo* like paracellular localization of the tight junction proteins claudin-5, occludin, ZO-1, and adherens junctional protein p120 catenin (Fig. 1c). Prior to each experiment, the transendothelial electrical resistance (TEER) was measured, showing TEER values of 2485 Ω cm2, ± 80 (Fig. 1b).

### TfR expression in BECs

**Fig. 2:**
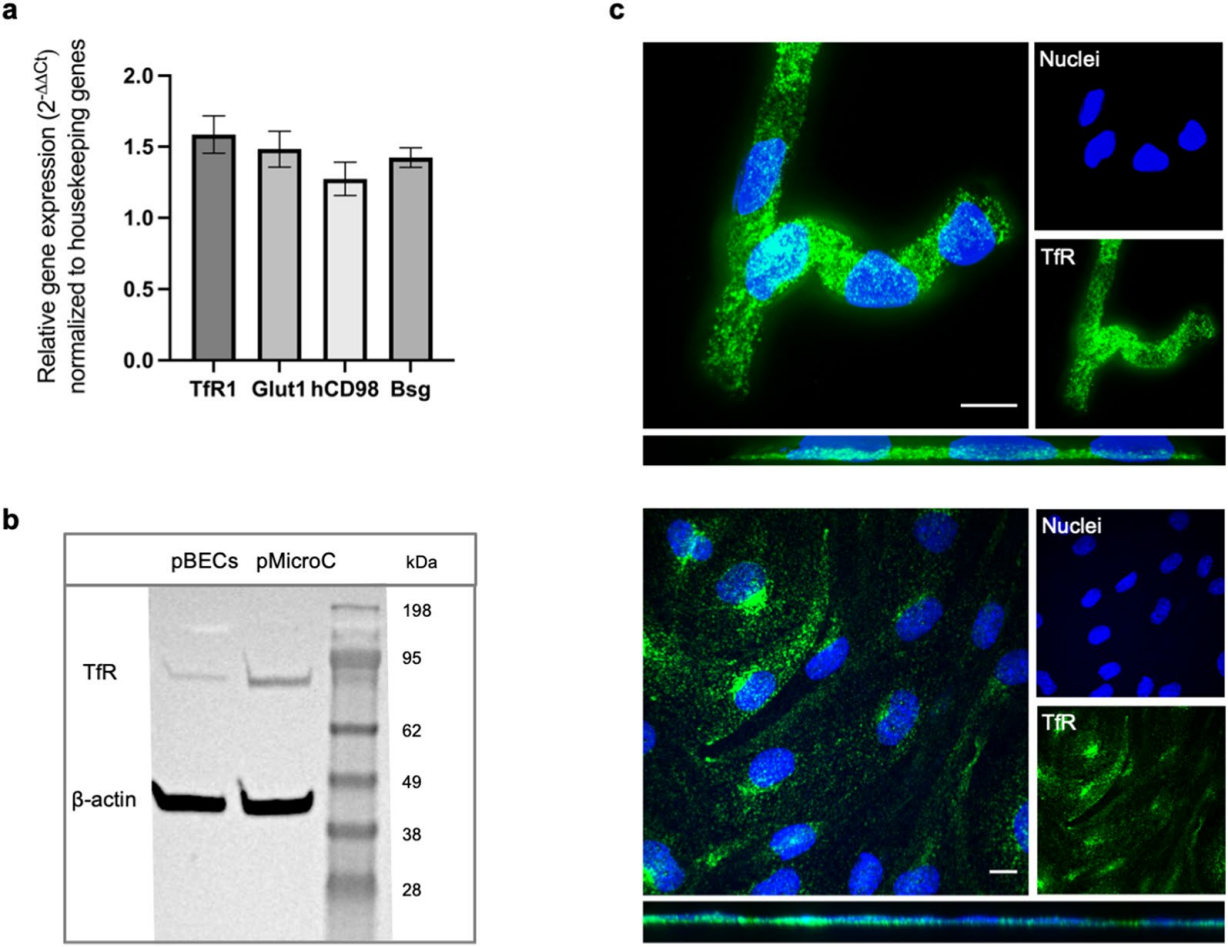
TfR expression in brain endothelial cells. Expression of the transferrin receptor and other receptor targets in the applied in vitro model was investigated by qPCR (**a**) with graph showing the relative mRNA gene expression levels (2−ΔΔCt), with data normalized to the housekeeping genes β2-microglobulin, Ribosomal Protein L4, TATA-Box Binding Protein, and Hypoxanthine Phosphoribosyl- transferase 1. TfR protein level expressions were investigated by Western blotting (**b**) using anti-TfR and ß-actin antibody on pBEC and pMicroC lysate. Normal IF staining and confocal microscopy of pMicroC and NCC established pBECs furthermore revealed TfR expression (green), with Hoechst stain of nuclei (blue) (**c**). Scale bar is equal to 10 μm

TfR expression on mRNA level in primary BECs was confirmed by qPCR analysis, revealing 1.6 ± 0.5 times the expression level of housekeeping genes, as presented in the graph showing the relative mRNA gene expression levels(2^−ΔΔCt^) (Fig. 2a). The fold-difference of the TfR expression was found to be similar to Glut1 expression (1.5 ± 0.5), whereas other newly suggested receptor targets for RTM, i.e., hCD98 and basigin (Bsg) [4] receptor were also expressed to a higher degree than housekeeping genes (1.3 ± 0.5 and 1.4 ± 0.3, respectively). TfR expression on protein level in porcine microcapillaries (pMicroC) and cultured pBECs was verified by Western Blotting (Fig. 2b), confirming presence in both lysates, however with a stronger signal for the microcapillary lysate. Additionally, normal IF and confocal microscopy of cultured porcine brain microcapillaries (Fig. 2c, upper), and BECs established in NCC with astrocytes (Fig. 2c, lower) confirmed TfR expression (green).

### Apicobasal polarization in BECs

**Fig. 3:**
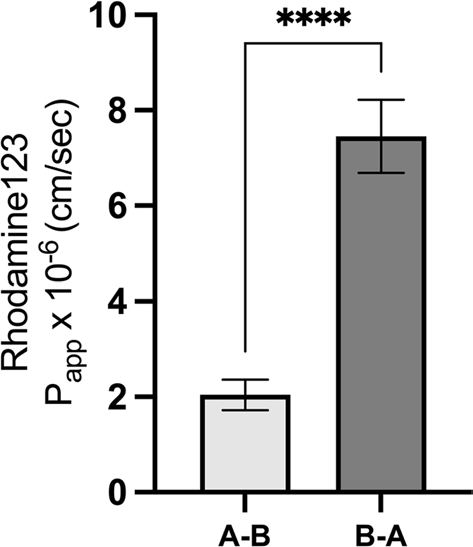
Validation of the in vitro model polarization by directional Rhodamine 123 transport. The apical-to-basal **A**, **B** and basal-to-apical **B**, **A**transendothelial permeability of two concentrations of the P-glycoprotein substrate Rhodamine 123 (3 and 30 µM) was assessed in the applied in vitro BBB model. The apparent permeability (Papp) for **B**, **A** transport was significantly higher than for **A**, **B** transport for both concentrations, indicating efflux pump activity and apicobasal polarity for the efflux transporter. Data are presented as mean values (± SEM). Significance was tested using t-test

Prior to studying apical and basal membrane domain localization and intracellular trafficking of TfR, establishment of apicobasal polarity of the applied in vitro BBB model was investigated. The bi-directional transport; apical-to-basal (A-B), and basal-to-apical (B-A), of the fluorescent tracer dye R123 with specificity for the ABC efflux transporter P-glycoprotein (P-gp, ABCB1) was performed using both a low and high concentration (3 μM and 30 μM). We found pBECs to show high efflux transport of R123, with P_app_ in the B-A direction (P_app_ B-A) being significantly higher (7.33 × 10^− 6^ ± 2.00 cm/sec (mean ± SD)) than in the A-B direction (P_app_ A-B) (1.94 × 10^–6^ ± 1.01 cm/sec) (Fig. 3). The resulting mean efflux ratio, calculated from P_app_(B-A)/P_app_(A-B) from each experiment was 3.99 ± 1.16. With this apicobasal polarization of the model, we continued the studies of the TfR apicobasal polarization.

### Apical and basal TfR membrane localization in BECs

**Fig. 4:**
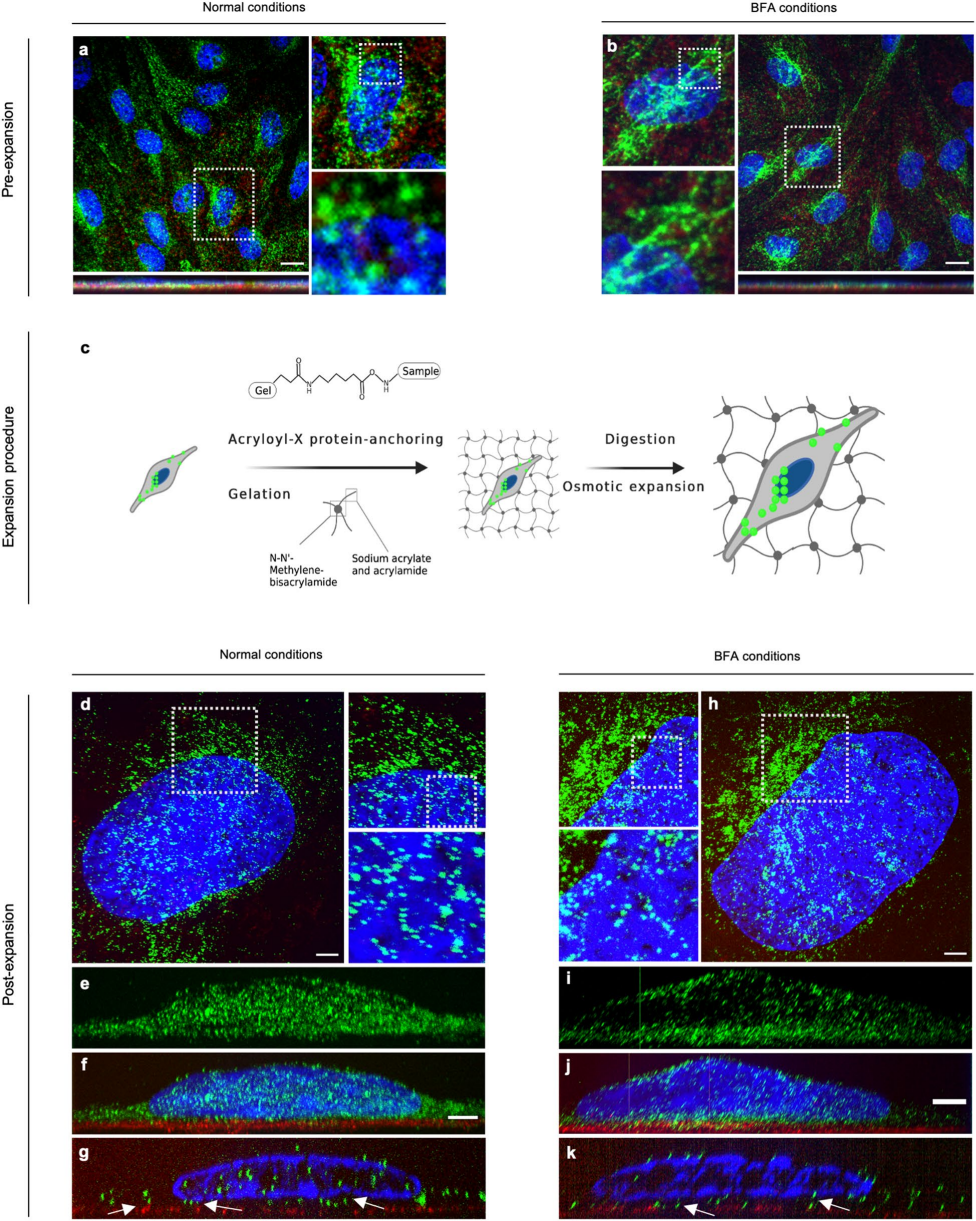
Apical and Basal TfR membrane domain localization in Brain Endothelial Cells using Expansion Microscopy. Normal immunofluorescence (IF) confocal micrographs (**a**, **b**, pre-expansion) served to control for sufficient isotropic expansion (**c**) and staining pattern of expansion specimens (**d**–**k**). Representative pre-expansion IF images show BECs established on collagen IV pre-coated filter membranes in non-contact co-culture (NCC) with astrocytes under normal and retended endoplasmic-Golgi trafficking conditions from Brefeldin A (BFA) treatment (**c**) with images presented as maximum intensity z-projections of nuclei (blue), collagen IV (red) and TfR (green) stainings, obtained by confocal microscopic imaging with 60 × magnification (UPlanSApo 60X, NA 1.20, water objective lens). Top view illustrates the rough overview of TfR distribution patterns while the below orthogonal view illustrates the limitation of a detailed overview using the normal IF technique. Expansion Microscopy (ExM) allowed visualization of the detailed TfR localization, with confocal micrographs of expanded BEC specimens in orthogonal view (**e**–**g**, **i**–**k**) showing nuclei staining (blue), collagen IV staining (red) marking the basal membrane localization, and TfR staining (green). Representative single slides present the capability of differentiating the apical and basal membranes and visualize TfR distribution, with white arrows marking basal TfR. Scale bar is equal to 10 μm for micrograph **a–c** and 1.6 μm for micrograph **d**–**k**

To elucidate contradictory beliefs of TfR trafficking in BECs [2] and contribute to the limited investigations of TfR localization in the basal domain, we continued the studies with confocal microscopy and ExM of the TfR apicobasal polarization. Using normal IF staining and confocal imaging of NCC established pBECs (Fig. 4a, b, pre-expansion), TfR expression (green) could be confirmed. However, the orthogonal views (Fig. 4a + b, lower images) of these micrographs did not allow more precise localization of the fluorescence signals or resolution of the plasma membrane domains, with the basal membrane localization indicated from collagen IV staining (red). From the top view, the resolution allowed some patterns of TfR localization to be visualized, including perinuclear organization and patterns resembling vesicular localization. Furthermore, BFA conditions, inhibiting vesicular movement between ER and the Golgi apparatus was included to investigate whether retention of neosynthesized receptor protein would affect the membrane distribution and intracellular localization pattern. Here we observed intracellular TfR organizing in an elongated line or tubular formation (Fig. 4b), rather than a vesicular pattern as observed under normal conditions (Fig. 4a).

In order to differentiate receptor localization on the apical and basal plasma membranes, ExM was applied. By cross linking BEC specimens with acryloyl-X, acting as an anchor group for a following polyelectrolyte hydrogel expansion of the cells, and subsequently ensuring proper isotropic expansion by protease digestion, confocal imaging allowed visualization of a detailed and highly resolved localization of fluorescently labelled TfR (see Fig. 4c, for a schematic illustration of the expansion procedure). By physical expansion of the cellular specimens six times (see Additional file 3: Fig. S1), visual distinction between apical and basal membrane of the pBECs became possible (Fig. 4d–k).

Compared to the normal IF staining and confocal microscopy, the increased resolution in the ExM specimens revealed TfR trafficking to be highest in the perinuclear area, and to resemble line formations comparable to cytoskeletal structures, known to guide vesicles in cytoplasm via adaptor proteins. Examination of XY projections of the micrographs, creating an orthogonal view of the cellular specimens, clearly showed TfR to be distributed both intracellularly and at the two plasma membrane domains, thus confirming TfR to localize within the basal membrane domain (Fig. 4g + k, white arrows) under normal and BFA conditions.

**Fig. 5:**
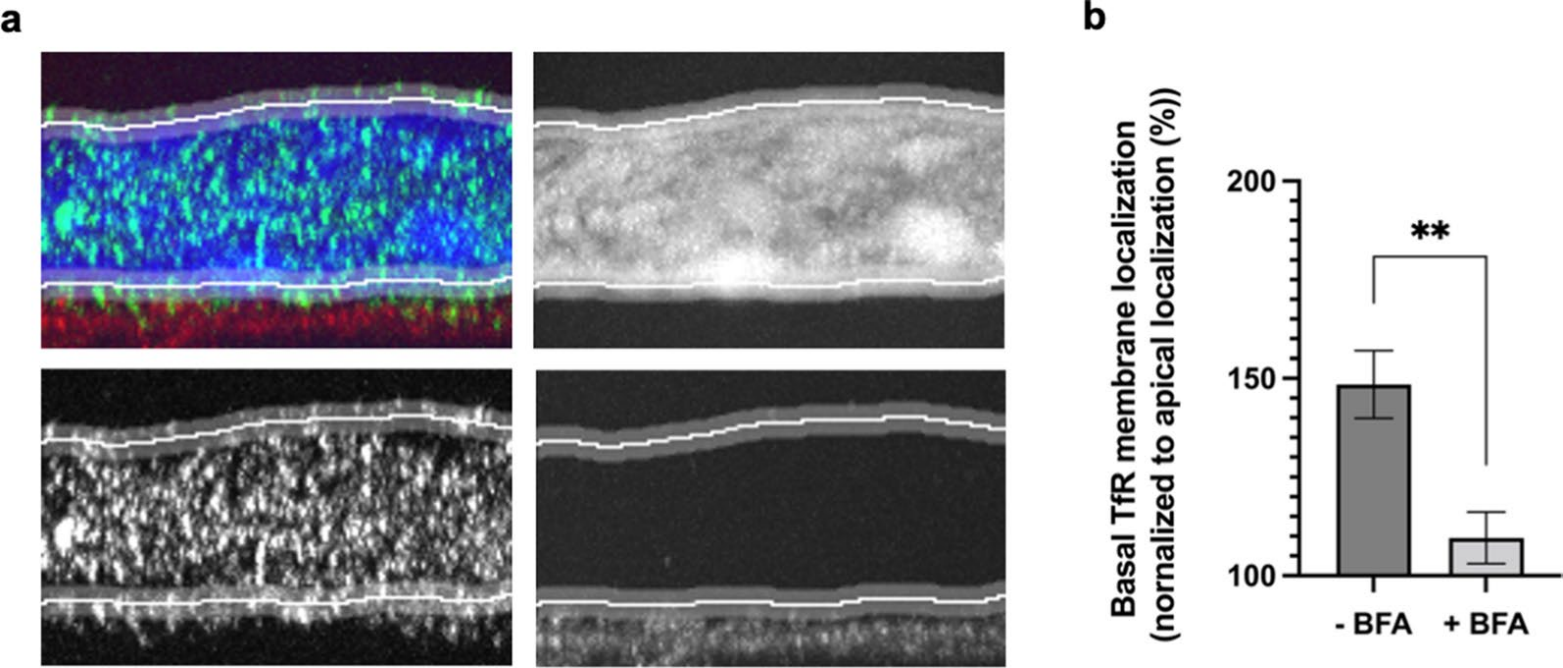
Quantification of Apical and Basal TfR membrane distribution. Quantification of TfR membrane localization based on Fiji line scans of the mean intensity of TfR signals in the apical and basal domains of BECs **a**, with graph **b** showing the basal membrane domain signals normalized to the apical membrane domain signals (%) under normal (- BFA) and BFA conditions (+ BFA). Statistical significance was tested using t-test

From line measurements of the fluorescence intensity within the two membranous domains (Fig. 5a), we found basal localization to constitute a large fraction of the TfR membrane localization when normalizing to the apical level Fig. 5b). Interestingly, BFA inhibition resulted in a significant change in the relative distribution on the apical and basal membrane, suggesting transport of the neosynthesized TfR to be involved (Fig. 5).

### Differentiating apical and basal TfR endosomal sorting

**Fig. 6:**
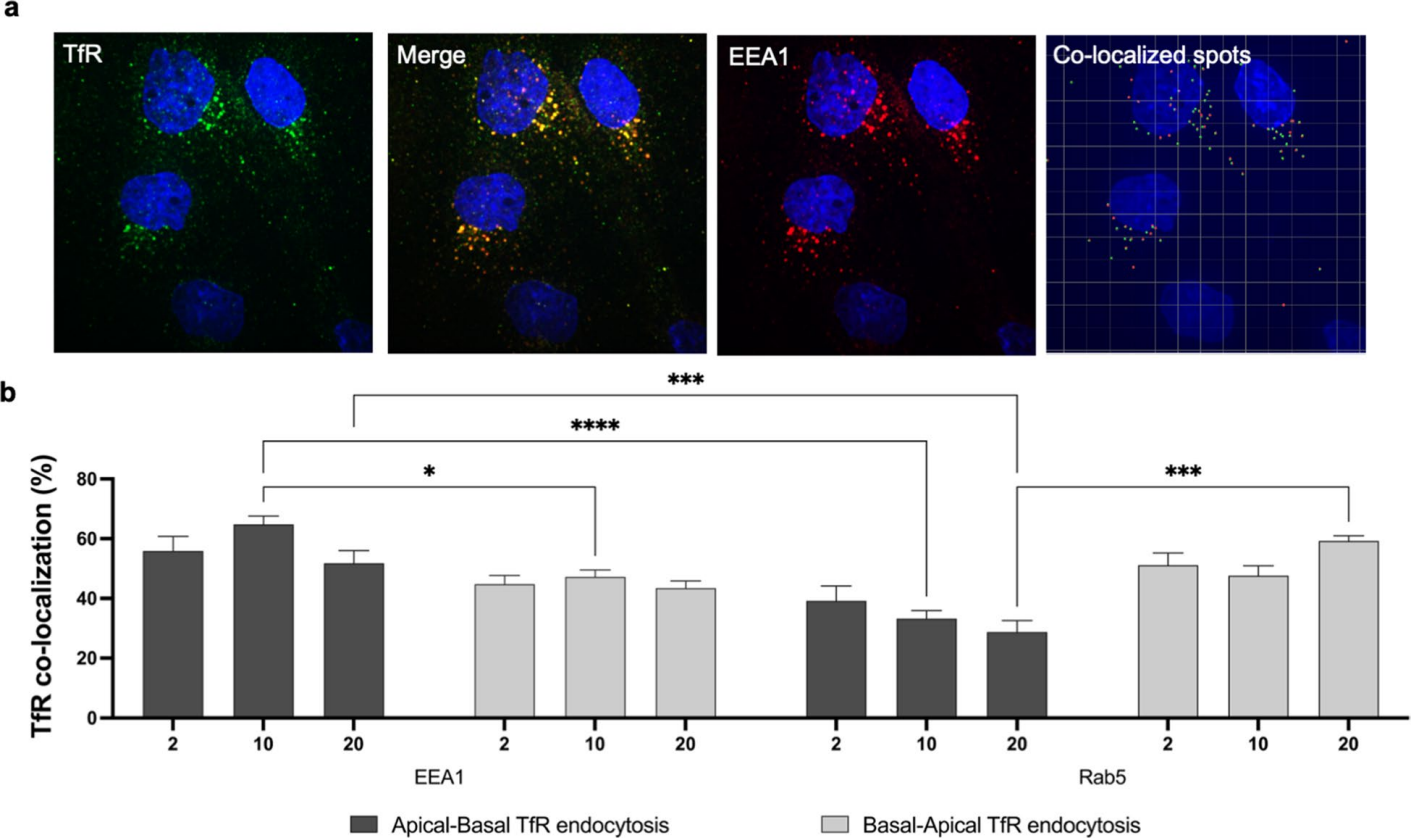
Immunofluorescence evaluation of TfR transcellular transport capability in BECs. Representative confocal images of apical and basal internalized TfR (green) and EEA1 (red) co-localization at 10 min **a**, analyzed using IMARIS spot segmentation and co-localization analysis. Hoechst stain of nuclei is blue. Graph shows the semi-quantification of TfR internalized from either the apical membrane (dark grey) or basal membrane (light grey), and co-localization with the markers of the intracellular compartments **b**. Significance was tested using two-way ANOVA with Tukey’s multiple comparison test

Using an immunofluorescence based, pulse-chase methodology for mapping receptor endocytosis and trafficking as previously described [39], we found the bi-directional endocytosis of TfR to significantly differientiate in co-localization with the intracellular markers EEA1 and Rab5 (Fig. 6). By saturating TfR on the apical or basal membrane of BECs with targeting antibody at low temperatures, and allowing endocytosis by re-incubation at 37 °C, 5% CO_2_, the receptor trafficking was chased following 2-, 10-, and 20 min incubation. Subsequent fixation, IF staining with intracellular markers and spot segmentation analysis revealed significant differences after 10 min. Here we found TfR endocytosed from the apical membrane to co-localization with EEA1 to a higher degree than Rab5 (****p < 0.0001). Furthermore, at 10 min TfR endocytosed from the apical membrane co-localized with EEA1 to a higher degree than TfR endocytosed from the basal membrane (*p < 0.05). After 20 min incubation, TfR endocytosed from the apical membrane still significantly differentiated in co-localization with EEA1 and Rab5 (***p < 0.001), and in Rab5 co-localization of TfR endocytosed from the basal membrane (***p < 0.001). Uptake form both sides further confirmed the apical and basal membrane localization of TfR found by ExM.

### Bi-directional apicobasal TfR transcellular transport capability in BECs

**Fig. 7:**
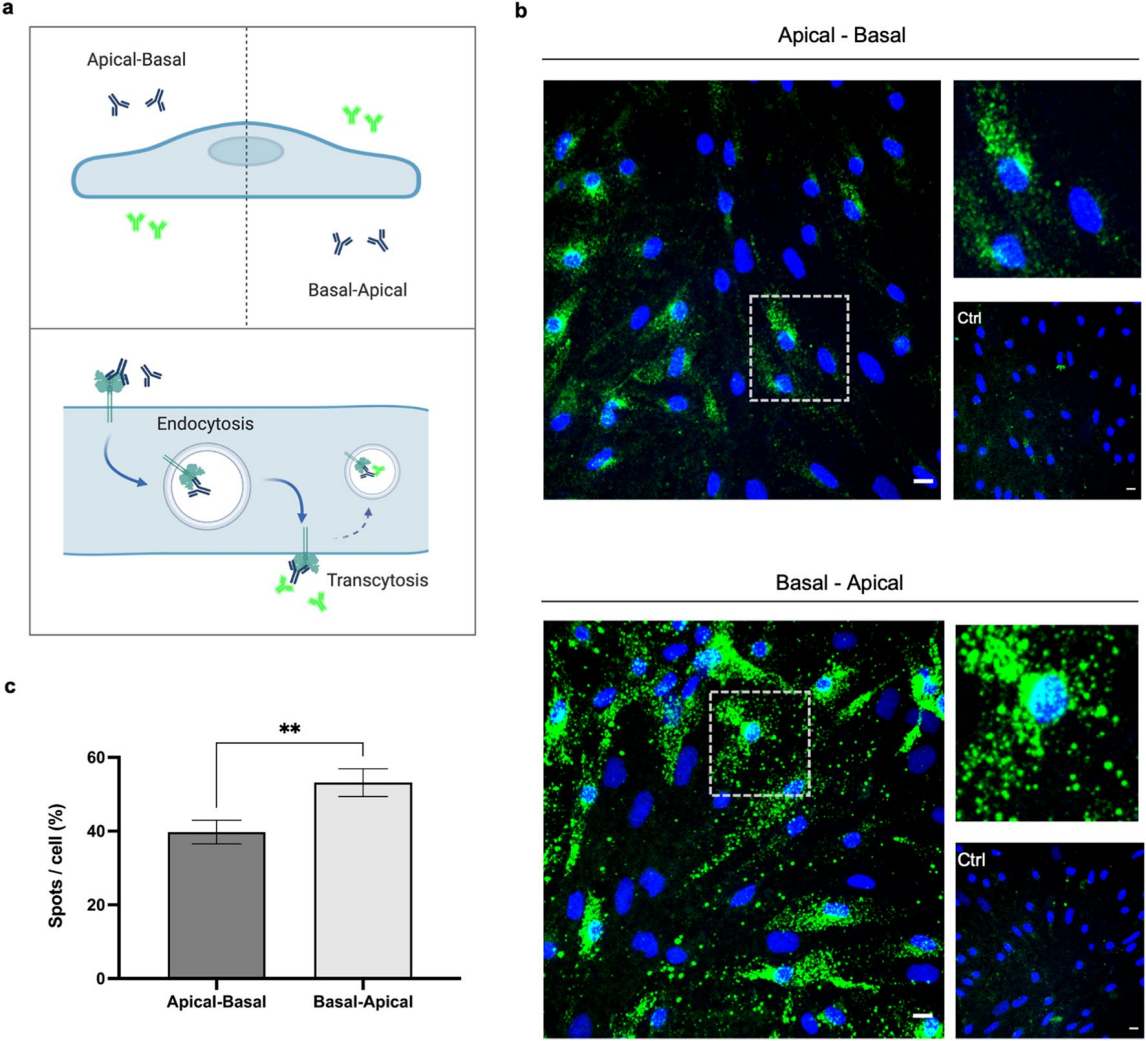
IF evaluation of TfR transcellular transport capability in BECs. Schematic illustration of the experimental approach for the receptor transcellular transport capability in the apical-to-basal (A-B) and basal-to-apical (B-A) direction using an immunocytochemistry assay **a**. Micrographs were obtained from confocal microscopy using 40 × magnification, **b**, with representative images showing the transcellular transport capability (green), control with secondary antibody only (Ctrl), and Hoechst staining of nuclei. Scale bar is equal to 10 μm. **c** Semi-quantitative analysis using spot detection, with data presented as spots/cell (5) normalized within each experimental replicate, and presented as mean ± SEM. Statistical significance was tested using t-test

Subsequent to the examination of apical and basal TfR membrane domain localization, we studied the capability of TfR transcellular transport in the A-B and B-A direction. As schematically illustrated (Fig. 7a), we applied primary anti-TfR antibody in either the upper or lower compartment of the in vitro Transwell setup, simultaneously with fluorescent secondary antibody in the opposite compartment. Using this previously applied setup for studying receptor trafficking [40, 41], primary antibody binding TfR on either the apical or basal membrane and trafficked to the adjacent plasma membrane were available to bind a fluorescent secondary antibody, detected by confocal microscopy. After 45 min of incubation, BECs were fixed and subsequently analyzed using confocal microscopy. In order to control for unspecific signal caused by trace of the secondary antibody, control specimens with only secondary antibody were also examined. Confocal micrographs revealed fluorescent signals in both directions (Fig. 7b), as well as differing patterns in the A-B and B-A directions, with limited trace from unspecific signal examined in the control with only secondary antibody (Ctrl). Findings of intracellular signals indicate transcellular trafficked TfR to internalize following transcellular trafficking, with a more homogenous, perinuclear pattern in the A-B direction and a more spread, heterogenous pattern in the B-A direction. A semi-quantitative analysis of the transcellular transport signals was performed using IMARIS spot detection, revealing that the number of spots pr. cell normalized within each experimental replicate showed significantly higher transport in the B-A than the A-B direction.

## Discussion

Investigation of the apicobasal polarity of BECs using fluorescent microscopy is subject to technical challenges with obtaining resolution good enough to enable differentiation of the close apical and basal membranes. This has limited previous investigations and could potentially also explain the contradictory findings of the protein and lipid distributions in apicobasal cell polarity in general [9, 11, 42]. Here, we examined the apicobasal polarity of TfR in the established BBB model, which previously exhibited polarized alpha-synuclein transport [43], and has been reported to provide influx and efflux transport, depending on the specific compound [44, 45]. To further validate the model, we examined the polarized transport of R123, a substrate for the transporter P-gp [35, 46], and considered an efflux transporter in BECs [47]. Here we found significant higher transport in the B-A direction than in the A-B, with an efflux ratio of approximately 4 (Fig. 2). In previous studies an efflux ratio > 2.5 is considered a verification of polarized efflux transport [48], thus confirming establishment of apicobasal polarity in pBECs of the applied model. This finding is in compliance with P-gp reported highly expressed in BECs [49–51], and resembling the in vivo findings of apicobasal polarity with P-gp efflux of CNS drugs [48]. Furthermore, this P-gp expression is utilized in the applied puromycin-treatment under the initial culturing of the brain microcapillaries, selectively favoring a culture of BECs, as previously described in detail [34, 52].

Microarray expression analysis of BECs and peripheral endothelial cells have shown TfR and the newly suggested target receptor basigin/CD147 (Bsg) to be highly enriched in BECs compared to endothelial cells of the liver and lung. Other popular targets such as insulin receptor (IR), low density lipoprotein-related protein 1 (LRP1), and newly suggested large amino acid transporter (LAT1/CD98) have shown similar expression in all the mentioned endothelial tissues [7, 53]. Examination of TfR expression on mRNA level from this study revealed TfR expression to be approximately 1.5 times higher than housekeeping genes, and likewise higher than Glut1 [54, 55], the primary transporter of glucose. Furthermore, Lat1/CD98 and Bsg did not exceed the expression of TfR (Fig. 3a), underscoring the high level of TfR expression in BECs.

With previous suggestions of basal localization of TfR and TfR trafficking from brain to blood, we wanted to study this phenomenon, since it in practice could diminish the level of drug delivery from blood to brain parenchyma, as basal localized TfR may carry back therapeutic constructs. We aimed to gain the necessary increased resolution for studying the plasma membrane distribution of TfR using expansion microscopy. This method can enable physical magnification of nanoscale structures, thus overcoming the limits of resolving features smaller than 300 nm using conventional optical imaging techniques. By optimizing the procedure of established protocols for ExM [37, 38], we managed to expand cells six times (Additional file 3: Fig. 1). The ExM specimens were investigated for incomplete homogenization and/or anisotropic expansion by including normal IF as a comparison control (Fig. 4a, b, pre-expansion), revealing labeling preservation and properly expanded specimens. In order to mark the basal membrane localization and having a navigator for the apicobasal orientation of the BECs and expanded specimens, we included Hoechst (nuclei) and collagen IV (collagen IV coated filter surface). Proper resolution of the pBEC membranes showed both apical and basal TfR membrane domain localization, with basal TfR localized slightly above the collagen IV staining (Fig. 4f, g + j, k).

Following protein synthesis and folding in ER, TfR is reported to traffic through the Golgi stacks to the trans-Golgi network (TGN) and endosomes in a signal-dependent manner [56, 57]. In the investigation of the TfR distribution under normal and BFA conditions, semi-quantitative measurements of the plasma membrane signals showed a significant change in the relative distribution under BFA conditions, with a reduced basal membrane domain signal (Fig. 5), suggesting this fraction to be supplied by neosynthesized TfR or a shift which favors transport to the apical membrane domain. From this data, transcellular transport capability of TfR cannot be excluded, since a basal fraction is still apparent under BFA conditions, and could arise from TfR undergoing apical to basal transcellular trafficking. It is noteworthy that numerous studies of the physiological dynamics of TfR trafficking and brain iron uptake point towards a mechanism involving apical endocytosis with subsequent endosomal iron dissociation from the TfR-Tf complex [2].

Anti-TfR antibodies have been found to accumulate in BECs and be trafficked towards lysosomal degradation with resultingly limited transcytosis. Re-engineering of the affinity for TfR has been attempted in order to improve the transcytosis, however requiring high doses to significantly increase the receptor occupancy [2, 58–61]. Interestingly, investigation of TfR endocytosis from the apical and basal membrane revealed differentiating co-localization with EEA1 and Rab5 (Fig. 6). A new interesting study, using quantitative CLEM for analyzing the localization of Rab5 and EEA1 in endo-lysosomal system, demonstrates that early Rab5 positive vesicles are in close proximity to the plasma membrane and direct the content in a fast-recycling direction, while the EEA1 positive endosome are directed towards the lysosomal degradation system [62]. If this mechanism also applies in polarized BECs, it would implicate that the basal endocytosed Rab5 positive vesicles would favor recycling, whereas the relative high fraction of TfR in EEA1 positive vesicles after apical endocytosis to a higher degree are sorted towards late endosomes and subsequent lysosomal degradation. Different anti-TfR antibodies may be sorted in such various ways, and this might explain why anti-TfR antibodies with low affinity and avidity have better transcytosis rates.

To elucidate if BECs support any transcytosis path for TfR specific antibodies, we applied our previously described IF assay to investigate transcellular receptor trafficking [40, 41]. The assay showed signal of transcytosed TfR antibody in both the A-B and B-A direction (Fig. 7). The included control setup with secondary antibody showed no signal (Fig. 0.7, ctrl.), excluding that the signal was caused by internalization of the secondary antibody itself. Semi-quantitative measurement of the signals showed significantly higher transcellular transport capability in the B-A than the A-B direction. Interestingly, these results indicate a TfR-driven trafficking path working to re-uptake TfR-directed ligands from the brain side of the BBB model. Even though these data demonstrate that there is a significant TfR-mediated efflux of anti-TfR in our in vitro porcine BBB model, the presences of TfR-expressing pericytes and astrocytes at the basal side could potentially compete with TfR in BEC and thereby reduce this observed efflux in favor of using TfR as a target for drug delivery.

In summary, the findings reveal that different trafficking paths are involved in the intracellular TfR trafficking, with a hitherto undescribed bi-directional apicobasal trafficking path, which could complicate the delivery of TfR based drug constructs to the brain parenchyma. Our data for the first time show basal TfR on resolved BEC plasma membranes. With results indicating transcellular transport capability of TfR, TfR trafficking not necessarily follow either transcellular transport or recycling paths, but may possibly be directed towards both transcellular and recycling paths. As BFA conditions affected the relative distribution of TfR on the apical and basal membranes, and mapping of the endosomal sorting to differentiate in co-localization with EEA1 and Rab5, different underlying mechanisms may control the transport from brain to blood and blood to brain. These findings are in accordance with the previous suggestion of TfR being administered in an apical, intracellular and basal pool, and potentially being a mechanism for regulation of brain iron concentrations, similar to what has been found for the iron trafficking in the gut [20].The presence of such regulatory system with TfR membrane localization being dynamically administered dependent on the brain iron concentration has been proposed as a mechanism for release of iron from the brain [20]. Importantly, this study has enabled confocal imaging resolution and confirmation of TfR on both the apical and basal plasma membranes of BECs with apicobasal bi-directional transport capability and thus potential ligand clearance from the brain, representing an understudied aspect of TfR-mediated brain drug delivery. Importantly, such elucidation and further improvements in of our understanding of the mechanisms involved in TfR trafficking in BECs are highly needed for unraveling the physiology of brain iron homeostasis in health and disease, as well as for optimizing and overcoming the current challenges for designing an effective brain drug delivery system.

## Supplementary Information

**Additional file 1: Table S1.** List of applied antibodies in the experiments.

**Additional file 2: Table S2.** Primers for qPCR analysis.

**Additional file 3: Figure S1.** Expansion factor. Measurements of the cell nuclei length (yellow line) from expanded (**a**) and pre-expanded BEC specimens (**b**) using a 60 times objective in confocal imaging and line measurements in FIJI, and plotted in a Box and whiskers plot (**c**) showing the minimum and maximum measurements in μm. Table (**d**) lists the mean values ± SD and the calculated expansion factor equal to the ratio of the pre-expanded and expanded specimens.

## Data Availability

Datasets applied for the studies are available from the corresponding author on reasonable request.

## Abbreviations

BBB: Blood–brain barrier
BEC: Brain capillary endothelial cell
BFA: Brefeldin A
CNS: Central-nervous system
EEA1: Early endosome antigen 1
ER: Endoplasmic reticulum
NCC: Non-contact co-culture
pBEC: Porcine brain endothelial cell
TEER: Transendothelial electrical resistance
RMT: Receptor-mediated transcytosis
Tf: Transferrin
TfR: Transferrin receptor

## References

[1] Duck KA, Connor JR (2016). Iron uptake and transport across physiological barriers. Biometals.

[2] Johnsen KB, Burkhart A, Thomsen LB, Andresen TL, Moos T (2019). Targeting the transferrin receptor for brain drug delivery. Prog Neurobiol.

[3] Nichols E (2022). Estimation of the global prevalence of dementia in 2019 and forecasted prevalence in 2050: an analysis for the global burden of disease study 2019. Lancet Public Heal.

[4] Pardridge WM (2022). A historical review of brain drug delivery. Pharmaceutics.

[5] Giugliani R (2021). Iduronate-2-sulfatase fused with anti-hTfR antibody, pabinafusp alfa, for MPS-II: a phase 2 trial in Brazil. Mol Ther.

[6] Freskgård PO, Urich E (2016). Antibody therapies in CNS diseases. Neuropharmacology.

[7] Zuchero YJY (2016). Discovery of Novel Blood-Brain Barrier Targets to Enhance Brain Uptake of Therapeutic Antibodies. Neuron.

[8] Toth AE, Holst MR, Nielsen MS (2020). Vesicular transport machinery in brain endothelial cells: what we know and what we do not. Curr Pharm Des.

[9] Worzfeld T, Schwaninger M (2016). Apicobasal polarity of brain endothelial cells. J Cereb Blood Flow Metab.

[10] Abbott NJ, Patabendige AAK, Dolman DEM, Yusof SR, Begley DJ (2010). Structure and function of the blood-brain barrier. Neurobiol Dis.

[11] Toth AE, Nielsen SSE, Tomaka W, Abbott NJ, Nielsen MS (2019). The endo-lysosomal system of bEnd. 3 and hCMEC/D3 brain endothelial cells. Fluids Barriers CNS.

[12] Abbott NJ, Rönnbäck L, Hansson E (2006). Astrocyte–endothelial interactions at the blood–brain barrier. Nat Rev Neurosci.

[13] Nakagawa S (2009). A new blood-brain barrier model using primary rat brain endothelial cells, pericytes and astrocytes. Neurochem Int.

[14] Armulik A (2010). Pericytes regulate the blood-brain barrier. Nature.

[15] Hawkins BT, Davis TP (2005). The blood-brain barrier/neurovascular unit in health and disease. Pharmacol Rev.

[16] Jefferies WA (1984). Transferrin receptor on endothelium of brain capillaries. Nature.

[17] Pardridge WM (1988). Recent advances in blood-brain barrier transport. Annu Rev Pharmacol Toxicol.

[18] Fishman JB, Rubin JB, Handrahan JV, Connor JR, Fine RE. Receptor-mediated transcytosis of transferrin across the blood-brain barrier. J Neurosci Res. 1987;18:299–304. 10.1002/jnr.490180206 3694713

[19] Moos T, Nielsen TR, Skjørringe T, Morgan EH (2007). Iron trafficking inside the brain. J Neurochem.

[20] Simpson IA (2015). A novel model for brain iron uptake: introducing the concept of regulation. J Cereb Blood Flow Metab.

[21] Morris CM, Keith AB, Edwardson JA, Pullen RGL (1992). Uptake and distribution of iron and transferrin in the adult rat brain. J Neurochem.

[22] Moos T, Morgan EH (2000). Transferrin and transferrin receptor function in brain barrier systems. Cell Mol Neurobiol.

[23] Taylor EM, Crowe A, Morgan EH (1991). Transferrin and iron uptake by the brain: effects of altered iron status. J Neurochem.

[24] Skjørringe T, Burkhart A, Johnsen KB, Moos T (2015). Divalent metal transporter 1 (DMT1) in the brain: Implications for a role in iron transport at the blood-brain barrier, and neuronal and glial pathology. Front Mol Neurosci.

[25] Chiou B, Connor JR (2018). Emerging and dynamic biomedical uses of ferritin. Pharmaceuticals.

[26] McCarthy RC, Kosman DJ (2013). Ferroportin and exocytoplasmic ferroxidase activity are required for brain microvascular endothelial cell iron efflux *. J Biol Chem.

[27] Moos T, Skjoerringe T, Gosk S, Morgan EH (2006). Brain capillary endothelial cells mediate iron transport into the brain by segregating iron from transferrin without the involvement of divalent metal transporter 1. J Neurochem.

[28] Burdo JR (1999). Cellular distribution of iron in the brain of the Belgrade rat. Neuroscience.

[29] Benkovic J, Connor JR (1993). Ferritin, transferrin and iron in normal and aged rat brains. J Comp Neurol.

[30] Piantino M (2022). Brain microvascular endothelial cells derived from human induced pluripotent stem cells as in vitro model for assessing blood-brain barrier transferrin receptor-mediated transcytosis. Mater Today Bio.

[31] Thomsen MS (2021). The blood-brain barrier studied in vitro across species. PLoS ONE.

[32] Huwyler J, Pardridge WM (1998). Examination of blood-brain barrier transferrin receptor by confocal fluorescent microscopy of unfixed isolated rat brain capillaries. J Neurochem.

[33] Tillberg PW, Chen F (2019). Expansion microscopy: scalable and convenient super-resolution microscopy. Annu Rev Cell Dev Biol.

[34] Nielsen SSE (2017). Improved method for the establishment of an in vitro blood-brain barrier model based on porcine brain endothelial cells. J Vis Exp.

[35] Ozgür B, Saaby L, Langthaler K, Brodin B (2018). Data demonstrating the challenges of determining the kinetic parameters of P-gp mediated transport of low-water soluble substrates. Data Br.

[36] Saaby L, Brodin B (2017). A critical view on in vitro analysis of p-glycoprotein (p-gp) transport kinetics. J Pharm Sci.

[37] Chen F, Tillberg PW, Boyden ES (2015). Expansion micrsocopy. Science.

[38] Chozinski TJ (2016). Expansion microscopy with conventional antibodies and and fluorescent proteins. Nat Methods.

[39] Holst MR, Nielsen SSE, Nielsen MS, Turksen K (2021). Mapping Receptor Antibody Endocytosis and Trafficking in Brain Endothelial Cells BT. Permeability Barrier: Methods and Protocols.

[40] Siupka P (2017). Bidirectional apical–basal traffic of the cation-independent mannose-6-phosphate receptor in brain endothelial cells. J Cereb Blood Flow Metab.

[41] Klinger SC (2016). Polarized trafficking of the sorting receptor SorLA in neurons and MDCK cells. FEBS J.

[42] Sivandzade F, Cucullo L (2018). In-vitro blood-brain barrier modeling: a review of modern and fast-advancing technologies. J Cereb blood flow Metab Off.

[43] Alam P (2022). Polarized α-synuclein trafficking and transcytosis across brain endothelial cells via Rab7-decorated carriers. Fluids Barriers CNS.

[44] Linz U (2015). Transport of treosulfan and temozolomide across an in-vitro blood–brain barrier model. Anticancer Drugs.

[45] Di Marco A (2019). Application of an in vitro blood-brain barrier model in the selection of experimental drug candidates for the treatment of huntington’s disease. Mol Pharm.

[46] Ozgür B, Saaby L, Langthaler K, Brodin B (2018). Characterization of the IPEC-J2 MDR1 (iP-gp) cell line as a tool for identification of P-gp substrates. Eur J Pharm Sci Off J Eur Fed Pharm Sci.

[47] Miller DS (2010). Regulation of P-glycoprotein and other ABC drug transporters at the blood-brain barrier. Trends Pharmacol Sci.

[48] Mahar Doan KM (2002). Passive permeability and P-glycoprotein-mediated efflux differentiate central nervous system (CNS) and non-CNS marketed drugs. J Pharmacol Exp Ther.

[49] Jetté L, Têtu B, Béliveau R (1993). High levels of P-glycoprotein detected in isolated brain capillaries. Biochim Biophys Acta.

[50] Cordon-Cardo C (1990). Expression of the multidrug resistance gene product (P-glycoprotein) in human normal and tumor tissues. J Histochem Cytochem.

[51] Fojo AT (1987). Expression of a multidrug-resistance gene in human tumors and tissues. Proc Natl Acad Sci.

[52] Perrière N (2005). Puromycin-based purification of rat brain capillary endothelial cell cultures. Effect on the expression of blood-brain barrier-specific properties. J Neurochem.

[53] Tam SJ (2012). Death receptors DR6 and TROY regulate brain vascular development. Dev Cell.

[54] Devraj K (2011). GLUT-1 glucose transporters in the blood-brain barrier: differential phosphorylation. J Neurosci Res.

[55] Pardridge WM, Boado RJ, Farrell CR (1990). Brain-type glucose transporter (GLUT-1) is selectively localized to the blood-brain barrier. Studies with quantitative western blotting and in situ hybridization. J. Biol. Chem.

[56] Futter CE, Connolly CN, Cutler DF, Hopkins CR (1995). Newly synthesized transferrin receptors can be detected in the endosome before they appear on the cell surface. J Biol Chem.

[57] Deffieu MS (2021). Rab7-harboring vesicles are carriers of the transferrin receptor through the biosynthetic secretory pathway. Sci Adv.

[58] Yu YJ (2011). Boosting brain uptake of a therapeutic antibody by reducing its affinity for a transcytosis target. Sci. Transl Med.

[59] Bien-Ly N (2014). Transferrin receptor (TfR) trafficking determines brain uptake of TfR antibody affinity variants. J Exp Med.

[60] Niewoehner J (2014). Increased brain penetration and potency of a therapeutic antibody using a monovalent molecular shuttle. Neuron.

[61] Johnsen KB (2018). Antibody affinity and valency impact brain uptake of transferrin receptor-targeted gold nanoparticles. Theranostics.

[62] van der Beek J, de Heus C, Liv N, Klumperman J (2022). Quantitative correlative microscopy reveals the ultrastructural distribution of endogenous endosomal proteins. J Cell Biol.

