# Correction to: Apicobasal transferrin receptor localization and trafficking in brain capillary endothelial cells

**DOI:** 10.1186/s12987-023-00452-1

**Published:** 2023-06-19

**Authors:** Simone S. E. Nielsen, Mikkel R. Holst, Kristine Langthaler, Sarah Christine Christensen, Elisabeth Helena Bruun, Birger Brodin, Morten S. Nielsen

**Affiliations:** 1 Department of Biomedicine, Faculty of Health, Aarhus University, Aarhus C, 8000 Denmark; 2 CNS Drug Delivery and Barrier Modelling, University of Copenhagen, Copenhagen, Denmark; 3 Translational DMPK, H. Lundbeck A/S, Copenhagen, Denmark; 4 Pathology & Imaging, Novo Nordisk, Måløv, Denmark; 5 Department of Pharmacy, Faculty of Health and Medical Sciences, University of Copenhagen, Copenhagen, 2100 Denmark

**Correction:** ***Fluids Barriers CNS*** **20, 2 (2023)**

10.1186/s12987-022-00404-1

In this article [1], the author name of Sarah Christine Christensen was unintentionally omitted.

The author list has been updated and the original article has been corrected.

The authors would like to apologise for any inconvenience caused.
