# Supplementary figures and images for: Apicobasal transferrin receptor localization and trafficking in brain capillary endothelial cells
Source: Fluids Barriers CNS. 2023 Jan 9;20:2. doi: 10.1186/s12987-022-00404-1 (PMC9830855; doi:10.1186/s12987-022-00404-1)

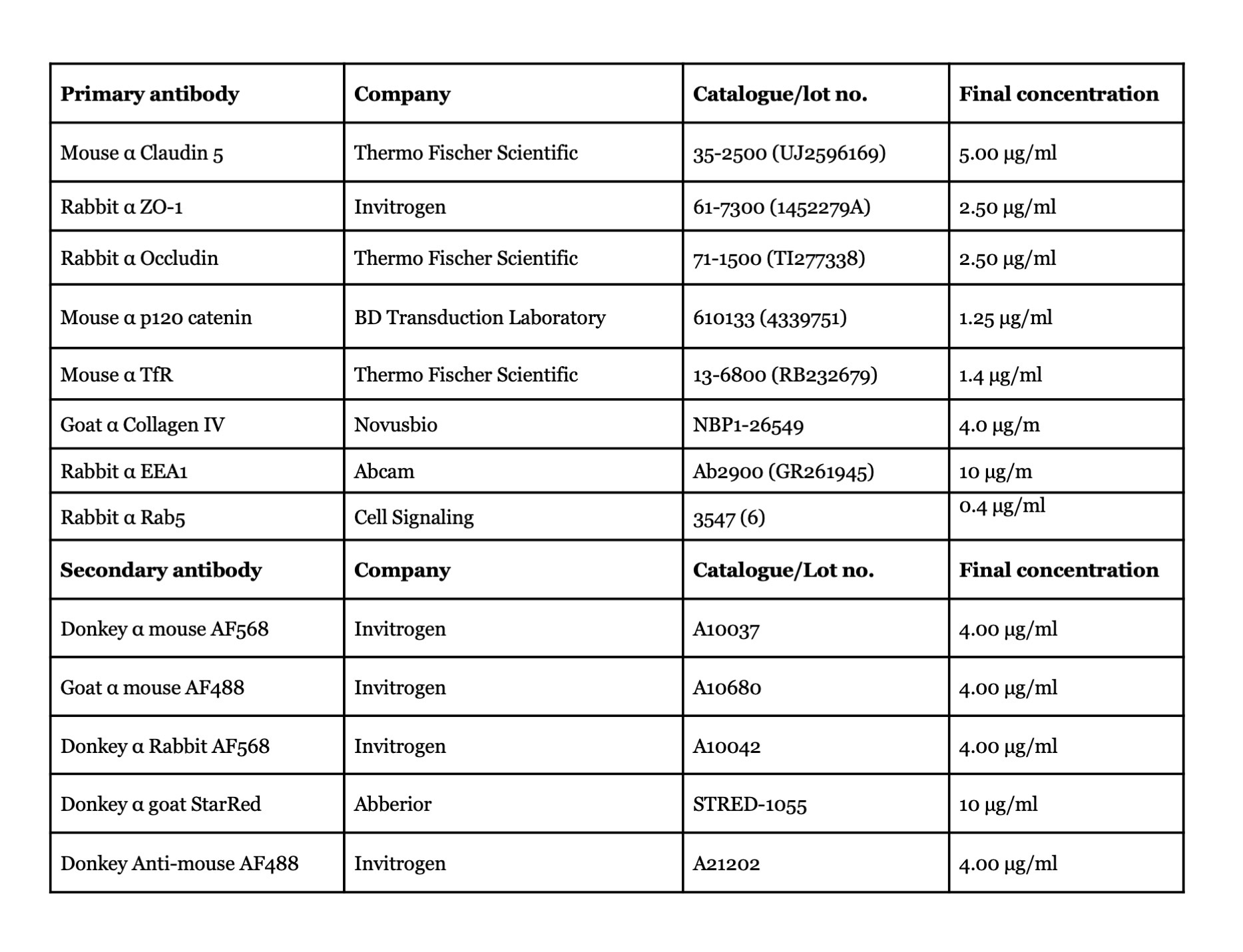

Supplement: Supplementary file 1 — Additional file 1: Table S1. List of applied antibodies in the experiments. [file 12987_2022_404_MOESM1_ESM.jpg]

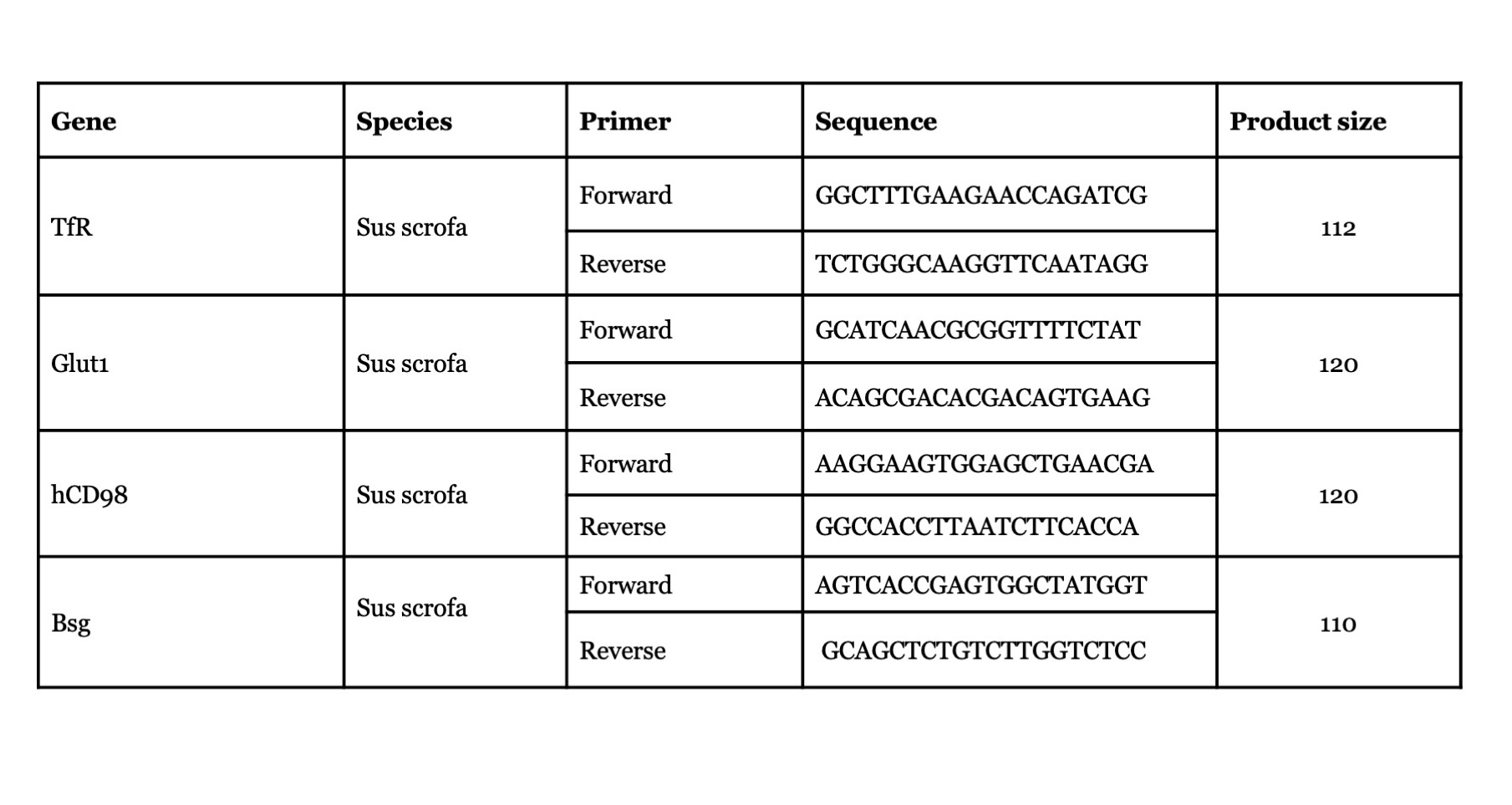

Supplement: Supplementary file 2 — Additional file 2: Table S2. Primers for qPCR analysis. [file 12987_2022_404_MOESM2_ESM.jpg]

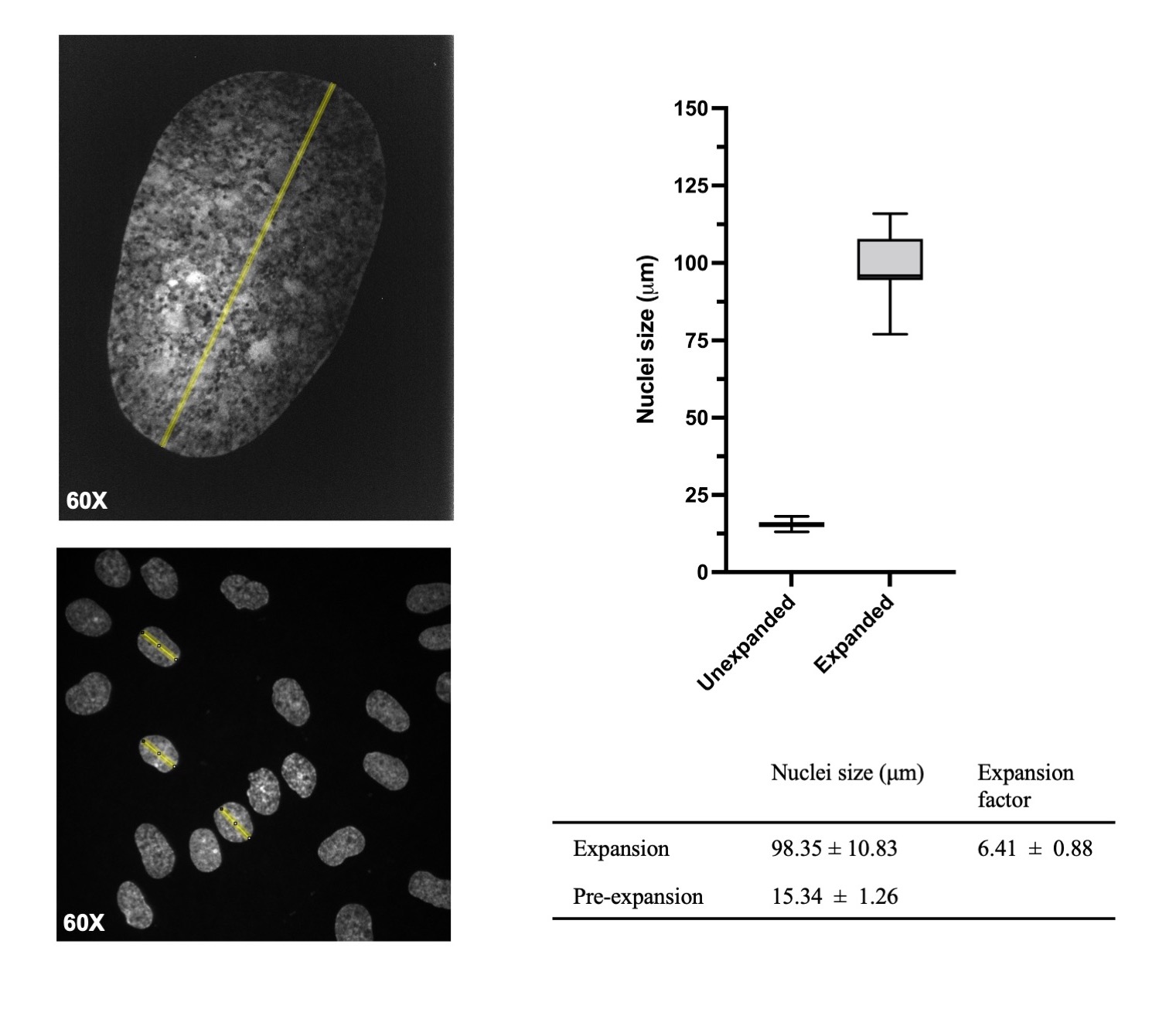

Supplement: Supplementary file 3 — Additional file 3: Figure S1. Expansion factor. Measurements of the cell nuclei length (yellow line) from expanded (a) and pre-expanded BEC specimens (b) using a 60 times objective in confocal imaging and line measurements in FIJI, and plotted in a Box and whiskers plot (c) showing the minimum and maximum measurements in μm. Table (d) lists the mean values ± SD and the calculated expansion factor equal to the ratio of the pre-expanded and expanded specimens. [file 12987_2022_404_MOESM3_ESM.jpg]
